# Autosomal Recessive Spastic Ataxia of Charlevoix-Saguenay (ARSACS) in a Thai Patient: The Classic Clinical Manifestations, Funduscopic Feature, and Brain Imaging Findings with a Novel Mutation in the *SACS* Gene

**DOI:** 10.5334/tohm.68

**Published:** 2020-06-08

**Authors:** Jindapa Srikajon, Yuvadee Pitakpatapee, Chanin Limwongse, Niphon Chirapapaisan, Prachaya Srivanitchapoom

**Affiliations:** 1 Division of Neurology, Department of Medicine, Faculty of Medicine, Siriraj Hospital, Mahidol University, Bangkok, TH; 2 Division of Medical Genetics, Department of Medicine, Faculty of Medicine, Siriraj Hospital, Mahidol University, Bangkok, TH; 3 Department of Ophthalmology, Faculty of Medicine, Siriraj Hospital, Mahidol University, Bangkok, TH

**Keywords:** Autosomal recessive spastic ataxia of Charlevoix-Saguenay, ARSACS, SACS gene, novel mutation, hereditary ataxia

## Abstract

**Background:**

A 38-year-old woman was diagnosed autosomal recessive spastic ataxia of Charlevoix-Saguenay (ARSACS) with a novel pathogenic variant in the *SACS* gene presented with gradually progressive spastic ataxia since the age of 2 years; then, she became wheelchair-bound at the age of 28 years.

**Phenomenology:**

The patient presented a combination of cerebellar dysfunctions e.g., gaze-evoked nystagmus, scanning speech, finger dysmetria, and wide-based gait, lower limb spasticity, and typical funduscopic examination which was a hypermyelinated nerve fibers radiating from the optic disc.

**Educational value:**

At present, ARSACS is recognized as a rare, worldwide, inherited movement disorder in which we should to aware of a diagnosis of this disorder in the patient who is presented with *FXN* gene negative early-onset spastic ataxia.

## Case summary

**Figure 1:**
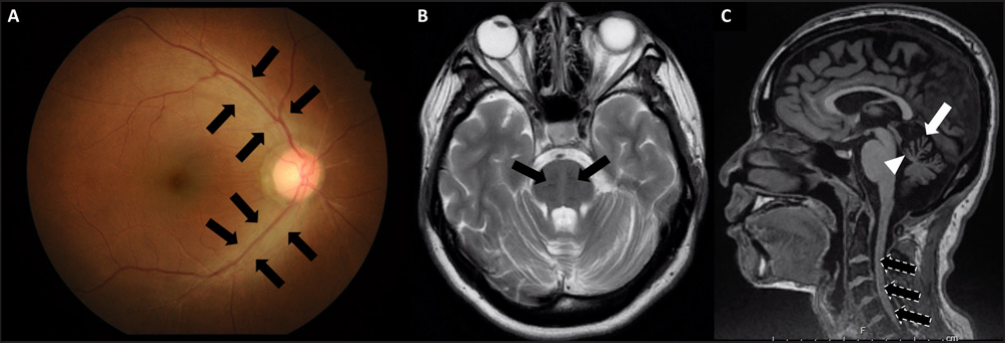
**Funduscopic and Neuroimaging findings of the patient. (1A)** Hypermyelinated nerve fibers radiating from the optic disc (black arrow); **(1B)** T2-weighted brain MRI showed multiple perpendicular linear hypointensities in pontine parenchyma (black arrow) and **(1C)** Superoanterior vermian (white arrow) and superior cerebellar peduncle (white arrow head) atrophy, as well as mild atrophy of the cervical spinal cord (black arrow with dash outline).

A 38-year-old woman presented with gradually progressive difficulty walking and frequent falls since the age of 2 years. At the age of 20 years, her gait was markedly unstable, and her speech was slurred. Finally, she became wheelchair-bound at the age of 28 years. Consanguinity was presented at the level of her paternal grandparents, but no family members were affected with neurological diseases. The ethnicity of the patient and her family were Thai. She and her family were originated from Cerebellar dysfunctions, including saccadic pursuit, hypermetric saccades, horizontal and vertical gaze-evoked nystagmus, scanning speech, finger dysmetria, and wide-based ataxic gait as well as spastic gait were presented (Video 1). Scleral telangiectasia, Kayser-Fleischer rings, oculomotor apraxia, and vertical supranuclear gaze palsy were not presented. Funduscopic examination showed hypermyelinated nerve fibers radiating from the optic disc (Figure 1A). Other neurological examination revealed normal cognitions and motor system with bilateral legs spasticity. Normal deep tendon reflexes except absent ankle reflexes with a positive Babinski sign were detected. Also, impaired proprioception of feet and ankles were presented.

**Video 1:** **Phenomenology of the patient. (Segment 1)** Saccadic pursuit, hypermetric saccades, horizontal and vertical gaze-evoked nystagmus; **(Segment 2)** Scanning speech, finger dysmetria; **(Segment 3)** Wide-based ataxic gait as well as spastic gait, and bilateral Pes cavus.

T2-weighted brain MRI showed multiple linear hypointensities in pontine parenchyma (Figure 1B). Superoanterior vermian and superior cerebellar peduncle atrophy, as well as mild atrophy of the cervical spinal cord, were shown (Figure 1C). Nerve conduction studies (NCS) showed sensorimotor demyelinating polyneuropathy. Autosomal recessive spastic ataxia of Charlevoix-Saguenay (ARSACS) was highly suspicious. Genetic testing for identifying the mutation of the *SACS* gene was performed by Invitae Corporation, CA, USA using a hybridization-based protocol and sequenced using illumine technology which is a target-capture exome sequencing. A homozygous, pathogenic variant, c.382_383del (p.Glu128Serfs*2), was identified in the *SACS* gene. The first documented case of ARSACS with a novel pathogenic mutation in Thailand was diagnosed.

## Discussion

This patient is the first reported case of ARSACS in Thailand. ARSACS is a hereditary movement disorder characterized by the classic triad, including progressive early-onset cerebellar ataxia, lower limb spasticity, and peripheral polyneuropathy [1]. It has the highest prevalence in northeastern Quebec, Canada. However, ARSACS has recently been reported in many countries outside Canada, for example, Germany, Japan, Tunisia, Italy, Netherland, and Brazil [2,3,4]. Clinical variations of non-Quebec patients have been reported, such as normal funduscopic examination or normal NCS [3]. However, our patient followed the typical manifestations of Quebec’s patient even she presented a novel mutation in the *SACS* gene. Pontine linear hypointensities in T2W sequence is a pathognomonic finding in this disease [2]. Cervical and thoracic spinal cord thinning as in our patient is occasionally reported1; nevertheless, it has never been reported in other non-Quebec cases. We should to aware of a diagnosis of ARSACS in the patient who is presented with *FXN* gene negative early-onset spastic ataxia.
